# Diagnostic differentiation between asthma and COPD in primary care using lung function testing

**DOI:** 10.1038/s41533-022-00298-4

**Published:** 2022-09-05

**Authors:** Jelle D. M. Bouwens, Erik W. M. A. Bischoff, Johannes C. C. M. in ’t Veen, Tjard R. Schermer

**Affiliations:** 1 Department of Primary and Community Care, Radboud Institute for Health Sciences, Radboud University Medical Center, Nijmegen, The Netherlands; 2 Canisius Wilhelmina Hospital, Nijmegen, The Netherlands; 3 Department of Pulmonary Medicine, ErasmusMC, Rotterdam, The Netherlands; 4 Department of Pulmonary Diseases, Franciscus Gasthuis & Vlietland, Rotterdam, The Netherlands; 5 Science Support Office, Gelre Hospitals, Apeldoorn, The Netherlands

**Keywords:** Asthma, Chronic obstructive pulmonary disease, Respiratory signs and symptoms, Epidemiology

## Abstract

Asthma and COPD are defined as different disease entities, but in practice patients often show features of both diseases making it challenging for primary care clinicians to establish a correct diagnosis. We aimed to establish the added value of spirometry and more advanced lung function measurements to differentiate between asthma and COPD. A cross-sectional study in 10 Dutch general practices was performed. 532 subjects were extensively screened on respiratory symptoms and lung function. Two chest physicians assessed if asthma or COPD was present. Using multivariable logistic regression analysis we assessed the ability of three scenarios (i.e. only patient history; diagnostics available to primary care; diagnostics available only to secondary care) to differentiate between the two conditions. Receiver operator characteristics (ROC) curves and area under the curve (AUC) were calculated for each scenario, with the chest physicians’ assessment as golden standard. Results showed that 84 subjects were diagnosed with asthma, 138 with COPD, and 310 with no chronic respiratory disease. In the scenario including only patient history items, ROC characteristics of the model showed an AUC of 0.84 (95% CI 0.78–0.89) for differentiation between asthma and COPD. When adding diagnostics available to primary care (i.e., pre- and postbronchodilator spirometry) AUC increased to 0.89 (95% CI 0.84–0.93; *p* = 0.020). When adding more advanced secondary care diagnostic tests AUC remained 0.89 (95% CI 0.85–0.94; *p* = 0.967). We conclude that primary care clinicians’ ability to differentiate between asthma and COPD is enhanced by spirometry testing. More advanced diagnostic tests used in hospital care settings do not seem to provide a better overall diagnostic differentiation between asthma and COPD in primary care patients.

## Introduction

Asthma and chronic obstructive pulmonary disease (COPD) are both common chronic respiratory diseases affecting approximately 1 in 12 people worldwide^1,2^. The two conditions are defined as different disease entities with unique pathophysiological mechanisms and characteristic clinical features^1,2^. The underlying pathophysiology in COPD is characterized predominantly by neutrophilic inflammation, whereas in asthma the inflammatory pattern is mostly due to eosinophilic inflammation^3^. Asthma typically presents with intermittent respiratory symptoms caused by airflow obstruction predominantly due to bronchial hyperresponsiveness^4^. Asthma is often presented at younger age as part of an atopic constitution, but can also be diagnosed in adulthood^1^. In contrast, COPD is a slowly progressive lung disease with patients having persistent respiratory symptoms and airflow obstruction^2^. In high-income countries like the Netherlands COPD usually presents in patients older than forty who are generally current or former smokers^2^. Patients with asthma or COPD are mostly diagnosed and managed by primary care clinicians.

Looking at the classic pathophysiological and clinical presentations, the distinction between asthma and COPD seems clear, but in clinical practice patients often show features of both diseases^5,6^. These similarities make it difficult for clinicians to distinguish between asthma and COPD^7^, especially in older and more diverse patient populations encountered in primary care^8–10^. However, differentiating between the two respiratory conditions is important as they have different pharmacotherapeutic regimens. In patients with asthma, inhaled corticosteroids (ICS) are highly effective in reducing symptoms and reducing the risk of asthma-related mortality^1^. In contrast, patients with COPD respond poorly to ICS and are mainly treated with (long-acting) bronchodilators to relieve symptoms^2^. In addition to this, misdiagnosing asthma for COPD could lead to serious health risks considering that monotherapy with long-acting bronchodilators is contra-indicated in asthmatics since it increases the risk of severe exacerbation^11–13^. On the other hand, (unnecessary) treatment with ICS may cause pneumonia and increased risk of osteoporosis^14–17^.

Thus, establishing a correct diagnosis is essential for optimal treatment of asthma and COPD, but this can be challenging for primary care clinicians. Supporting them in the diagnostic process seems therefore essential, but this also depends on the availability of diagnostic tools. Although quality spirometry has shown to be feasible in primary care settings^18^ there is substantial room for improvement of its use to accurately diagnose chronic respiratory diseases^19,20^. Thus, the first aim of our current study was to establish which patient characteristics distinguish between patients diagnosed with asthma or COPD. The second and main aim was to establish the added value of spirometry and more advanced lung function measurements to differentiate between these two chronic airways diseases.

## Methods

### Study design and population

In this observational multi-centre cross-sectional study, we compared patients diagnosed with asthma, patients diagnosed with COPD, and subjects without underlying chronic obstructive lung conditions using data from a previous study, i.e., the *Detection, Intervention and Monitoring of COPD’* (DIMCA) program^21^. This program was originally set up to improve early detection of chronic airways disease in general practices. A random sample of 1,749 adult subjects (20–70 years) from ten general practices in The Netherlands were invited to participate^21^. At the start of the program, patients with pre-existing asthma, COPD or another airway disease were excluded. In 2007, ten years after the start of the initial DIMCA program, all subjects (now aged 30–80 years) received an invitation for a comprehensive respiratory assessment consisting of extensive lung function measurements and a myriad of medical history questions^22^. A total of 532 subjects agreed to participate in this follow-up study. The results of the respiratory assessment of these subjects were submitted to two experienced chest physicians who assessed if a chronic airways disease (i.e., COPD or asthma) was present or absent using a standardized protocol^22^ that was based on the international clinical guideline criteria that applied at the time of the study (see below). The results of the chest physicians’ assessments were used as the golden standard in the current study.

The study was approved by the medical ethics review board CMO Regio Arnhem – Nijmegen (https://www.radboudumc.nl/over-het-radboudumc/kwaliteit-en-veiligheid/commissie-mensgebonden-onderzoek; file number: 2002/028). Participants provided written informed consent to take part in the study.

### Measurements

Study participants were instructed to interrupt the use of any bronchodilators they might use for a specified number of hours before their visit to the pulmonary function laboratory. Lung function testing involved pre- and postbronchodilator spirometry (both static and dynamic) and measurement of carbon monoxide diffusion capacity (DLCO) and bronchial hyperresponsiveness (BHR)^22^. Aerosolized salbutamol 800 µg and/or ipratropium 160 µg were used as bronchodilators and were administered by volume spacer. Postbronchodilator forced expiratory volume in one second (FEV1) was measured 15 min after salbutamol and 45 min after ipratropium. Bronchodilator reversibility was defined as an increase in FEV1 after bronchodilation by at least 12% and 200 mL. BHR was assessed by histamine challenge test and considered positive in case of a >20% drop in FEV1 at a provocative dose histamine of ≤8 mg/mL (PC20)^1,23^. All lung function tests were conducted by certified lung function technicians in a hospital-based pulmonary function laboratory and were performed in accordance with the 1994 American Thoracic Society standards^24^. Predicted normal lung function values for FEV1 were calculated using European Community for Coal and Steel reference values^25^. Following lung function testing, subjects were interviewed by the lung function technician regarding respiratory symptoms, smoking behaviour, presence of allergies and eczema, respiratory problems triggered by environmental exposures, and family history of COPD or asthma^22^.

### Diagnostic assessment

**Fig. 1:**
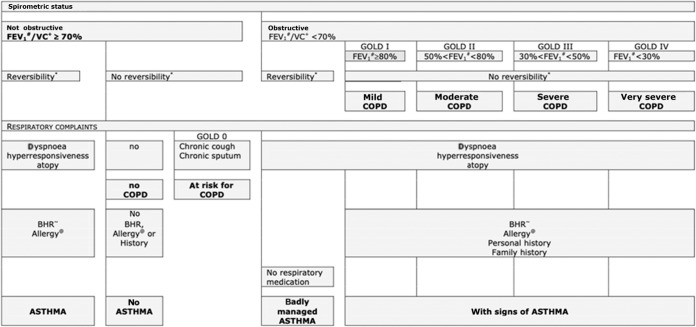
Decision tree used by the chest physicians to support their assessment of chronic lung disease diagnoses based on GOLD and GINA guidelines^22^. ^#^Postbronchodilator forced expiratory volume. ^+^Postbronchodilator vital capacity. *12% change in FEV1 (after bronchodilation), with a change of at least 200 mL. ~ Bronchial hyperresponsiveness (positive at a provocative histamine concentration ≤ 8mg/mL). ^@^Skin prick test.

**Fig. 2:**
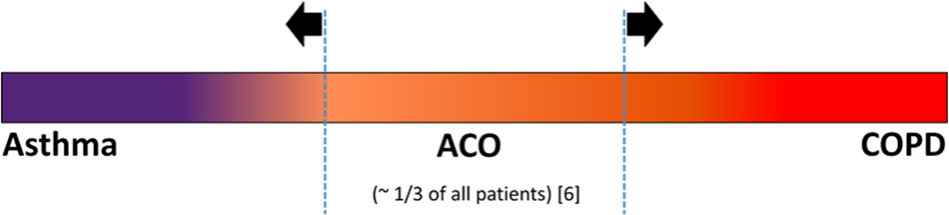
Schematic illustration of the spectrum of chronic obstructive airways disease diagnoses. The current study focusses on the parts to the left and right of the vertical dotted lines as indicated by the arrows. ACO asthma-COPD overlap, COPD chronic obstructive pulmonary disease.

Based on the results of the respiratory assessment the chest physicians assessed if a chronic airways disease (i.e., asthma or COPD) was present or absent using guideline criteria, their expert knowledge, and their clinical expertise^22^. Study subjects were randomly assigned to the chest physicians in a 1:1 ratio. If a subject was diagnosed with a chronic airways disease by the assigned chest physician the subject’s data was also presented to the other chest physician and a final joint diagnosis was established. To standardize the diagnostic process, a decision tree (Fig. 1) was created based on international clinical guideline criteria for diagnosing asthma (GINA guideline, 2007 update^26^) and COPD (GOLD guideline, 2006 update^27^) that applied at the time, in co-operation with the two chest physicians. In case of uncertainty about the respiratory diagnosis the chest physicians could request additional diagnostic tests (i.e., allergy skin testing, peak expiratory flow (PEF) monitoring) in order to maximize their diagnostic certainty^22^. Because the concept of asthma-COPD overlap (ACO) was introduced after the current study was conducted, the chest physicians did not consider a diagnosis of ACO as a part of their assessment. They were instructed to, based on their systematic assessment of all diagnostic information available, assign one single preferred diagnosis (i.e., either asthma or COPD) that fitted best according to their expert opinion. Figure 2 illustrates the spectrum of chronic obstructive airways disease diagnoses and the parts of the spectrum on which the current study focuses. Strictly for the purpose of describing the study population and its diagnostic subgroups (see Table 1) the Global Lung function Initiative (GLI) reference equations were applied at the time of the data analysis for the current paper^28^.

**Table 1:** Clinical features and lung function values of patients diagnosed with asthma and patients diagnosed with COPD.

	Chronic airways disease			No chronic airways disease
	Asthma	COPD	*p*-value	
*n* (% of total)	84 (16)	138 (26)		310 (58)
Demographic characteristics				
Age				
Mean (SD)	52.0 (11.4)	57.8 (10.0)	<0.001	54.4 (10.5)
Median (IQR)	49.8 (14.6)	57.0 (15.2)	<0.001	53.5 (14.2)
Range (youngest, oldest)	36.6 78.9	36.9 80.5		36.2, 80.7
Gender (% female, *n*)	59.5 (50)	44.2 (61)	0.027	56.1 (174)
BMI (mean, SD)	27.4 (4.3)	26.7 (4.0)	0.22	26.8 (4.0)
Smoking behaviour				
Ever smoking (%, *n*)	56.0 (47)	81.0 (111)	<0.001	65.5 (203)
Current smoking (%, *n*)	17.9 (15)	39.1 (54)	0.001	18.1 (56)
Packyear (mean, SD)	8.9 (14.4)	21.3 (19.5)	<0.001	10.8 (14.2)
Atopy (%, *n*)				
Ever allergy^a^	70.2 (59)	19.6 (27)	<0.001	7.7 (24)
Ever eczema	26.2 (22)	26.1 (36)	0.99	16.1 (50)
Hyperresponsiveness (%, *n*)				
Respiratory symptoms triggered by cold air smoke or (exhaust)fumes	71.4 (60)	59.4 (82)	0.071	22.6 (70)
Family history^b^ (%, *n*)				
Asthma	19.0 (16)	15.9 (22)	0.32	11.9 (37)
COPD	29.8 (25)	36.2 (50)	0.65	17.1 (53)
Current respiratory medication^c^ (%, *n*)				
Bronchodilator(s)	20.2 (17)	16.7 (23)	0.502	2 (0.6)
Inhaled corticosteroid	13.1 (11)	9.4 (13)	0.392	0
Respiratory symptoms (%, *n*)				
Cough^d^	20.2 (17)	26.1 (36)	0.32	4.5 (14)
Wheeze^e^	46.4 (39)	27.5 (39)	0.006	4.9 (17)
Phlegm^f^	11.9 (10)	19.6 (27)	0.14	3.9 (12)
Breathlessness^g^	40.5 (34)	30.4 (42)	0.13	4.8 (15)
Spirometry:				
PostBD FEV1/FVC (mean, SD)	74.2 (4.9)	63.3 (6.3)	<0.001	75.1 (8.0)
PostBD FEV1/FVC < 0.70 (%, *n*)	15.7 (13)	97.8 (135)	<0.001	13.6 (42)
PostBD FEV1 % predicted ECCS (mean, SD)	98.9 (13.9)	88.2 (16.2)	<0.001	107.1 (14.0)
PostBD FEV1 % predicted GLI^h^ (mean, SD)	91.8 (16.2)	81.9 (18.2)	<0.001	98.8 (17.9)
Reversibility (%, *n*)				
ΔFEV1 > 12% and >200 ml after BD	9.5 (8)	10.9 (15)	0.75	1.0 (3)
ΔFEV1 > 15% and >400 ml after BD^i^	7.2 (6)	2.9 (4)	0.18^j^	0 (0)
Other lung function test				
RV/TLC % (mean, SD)	32.2 (8.8)	35.4 (8.1)	0.005	31.0 (6.6)
Bronchial hyperresponsiveness^k^ (%, *n*)	45.2 (38)	42.8 (59)	0.68	5.5 (17)
Diffusion capacity^l^ (mean, SD)	8.5 (2.2)	7.6 (3.0)	0.016	8.6 (2.2)

*p*-values are for the comparison between the two diagnostic subgroups. Data of patients with no chronic airways disease as presented in the table serve as a general reference, but were not part of the current analysis.

*ECCS* European Community of Coal and Steel, *GINA* global initiative for asthma, *GLI* global lung function initiative, *LLN* lower limit of normal based in GLI prediction equations, *RV* residual volume, *SD* standard deviation, *TLC* total lung capacity.

^a^Allergic to pollen, animals, dust mites or seasonal symptoms.

^b^First degree relatives.

^c^As prescribed by the patient’s general practitioner and/or pulmonologist.

^d^Chronic cough in winter.

^e^Wheeze with or without breathlessness (in previous 12 months).

^f^Phlegm after getting out of bed (in previous 12 months).

^g^Breathlessness on exertion (in previous 12 months).

^h^Based on GLI reference equations (http://gli-calculator.ersnet.org/index.html). The % predicted FEV1 values as considered by the two chest physicians in the study were based on the 1993 ECCS reference equations. The GLI-based % predicted FEV1 values were not used by the two chest physicians.

^i^GINA (2021) states that confidence regarding presence of bronchodilator reversibility is greater if the increase is >15% and >400mls (1).

^j^Fisher’s exact test because one cell had an expected count <5.

^k^Decrease in FEV1 by >20% at provocative dose histamine of ≤8 mg/ml (PC20).

^l^Diffusion capacity in mmol/kPa/mi.

### Categorization of variables

In the present study, we categorized all items of the respiratory assessment in three subsections based on their availability in different healthcare settings, i.e., public health, primary care, and secondary care (Table 2). Subsection 1 consists of items that are available in any public health or healthcare setting since they require no measurements or testing equipment but only medical history questions (i.e., respiratory symptoms, smoking behaviour, body mass index (BMI)). Subsection 2 contains lung function test results that are available to primary care clinicians (i.e., spirometry and reversibility testing) in countries with well-developed healthcare systems^29–31^. Finally, Subsection 3 contains results from more advanced diagnostic tests as performed mainly in lung function laboratories in hospital care settings. These tests include measurement of static lung volumes, diffusion capacity, and histamine challenge testing.

**Table 2:**
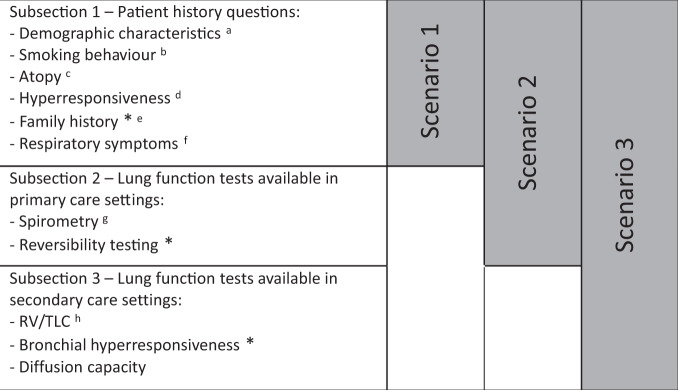
Categorization of variables in three subsections based on diagnostic availability and multivariable logistic regression analysis for the three scenarios.

^a^Age, gender and BMI.

^b^Ever and current smoking, packyears.

^c^Ever allergy, ever eczema.

^d^Respiratory symptoms triggered by cold air, smoke or (exhaust) fumes.

^e^First degree relative with asthma or COPD.

^f^Cough, wheeze, phlegm, breathlessness.

^g^Postbronchodilator FEV1 and FEV1/FVC.

^h^Residual volume/total lung capacity.

*Not included in multivariable logistic regression as *p* was <0.20 in univariate analysis.

### Statistical analysis

Demographic characteristics, clinical features and lung function values were univariately compared between the subgroups of patients diagnosed with asthma and COPD using independent *t*-tests and Chi-square tests. The further analysis focussed on assessing the ability to differentiate between these chronic obstructive lung diseases in different healthcare settings. Since physicians are not limited to asking a single medical history question or to conducting a single diagnostic test, we used multivariable logistic regression analysis to construct predictive models based on the data of the subjects who were diagnosed with asthma or COPD by the chest physicians (i.e., the binary outcome measure for this analysis was to have a diagnosis of asthma or a diagnosis of COPD). As described above, the items from the patient assessment were categorized in three subsections based on diagnostic availability and multivariable logistic regression models were run for three ‘scenarios’ (Table 2). In the first scenario, we only used the medical history items from Subsection 1 in the model. In the second scenario, we added diagnostic items available to primary care clinicians (i.e., Subsections 1 plus 2) to the model. In the third scenario, we added diagnostic items available to secondary care clinicians to the model (i.e., Subsections 1 plus 2 plus 3). Only items with a *p*-value ≤0.20 in the univariate analysis were considered relevant as predictors and were included in the respective models. In each scenario, the item with the highest *p*-value was manually removed from the model after which the logistic model was re-run (‘backward selection’). This step was repeated until only variables with *p*-values < 0.10 remained in the model for each scenario. Odds ratios for diagnosing asthma were calculated with COPD as reference group and vice versa. For each scenario a receiver operator characteristics (ROC) curve was created and the percentage explained variance (Nagelkerke R square) determined. Area under the curve (AUC) values from the ROC curves of the three scenarios were statistically compared using a non-parametric approach for correlated ROC curves^32^. SPSS statistics version 25.0 and SAS version 9.4 were used for the analyses. Missing data were not imputed. Two-sided *p*-values < 0.05 were considered statistically significant, except for the testing of the AUC values between Scenarios 1 and 2 and Scenarios 2 and 3, respectively, in which multiple testing was taken into account by using *p* < 0.025 to define statistical significance (i.e., Bonferroni correction: *p* = 0.05/2 = 0.025).

### Reporting summary

Further information on research design is available in the Nature Research Reporting Summary linked to this article.

## Results

### Study population

In the total sample of 532 study subjects (all Caucasians), 84 (16%) were diagnosed with asthma, 138 (26%) were diagnosed with COPD, and in 310 subjects (58%) no chronic airways disease was diagnosed (Table 1). Compared to patients with COPD the patients diagnosed with asthma were significantly younger (mean age 50.2 (SD 11.4) versus 57.8 (SD 10.0); *p* < 0.001) and more likely to be female (59.5% versus 44.2%; *p* = 0.027). There was no statistically significant difference in BMI between the two diagnostic subgroups (*p* = 0.22).

### Differences and similarities in clinical features and lung function

Table 1 gives an overview of the differences and similarities in demographic characteristics, clinical features and lung function values between patients with asthma and patients with COPD. Patients diagnosed with COPD were significantly more likely to be former or current smokers and had more packyears compared to patients with asthma (21.3 (SD 19.5) versus 9.1 (14.4); *p* < 0.001). Patients with asthma were significantly more likely to have allergies compared to patients with COPD (*p* < 0.001) but there was no difference in the prevalence of eczema between the subgroups (*p* = 0.99). Patients with asthma had significantly more often symptoms of wheezing (*p* = 0.006) compared to patients with COPD. The prevalence of having chronic cough, phlegm or breathlessness was not significantly different between the groups. Patients with COPD had significantly lower % predicted postbronchodilator FEV1 values (88.2% *versus* 98.9%; *p* < 0.001) compared to patients with asthma. There were no differences in the presence of reversibility (*p* = 0.75) or bronchial hyperresponsiveness (*p* = 0.68) between the two subgroups. No additional diagnostic tests were requested by the two chest physicians.

### Differentiating ability of diagnostic items

Demographic characteristics, clinical features and lung function tests yielded a total of 21 diagnostic variables (Table 1). Excluding items with *p*-values of >0.20 in the univariate analysis resulted in twelve items that were considered as relevant discriminants to be entered in the multivariable logistic models: age, gender, packyears, wheeze, phlegm, breathlessness, allergy, respiratory symptoms triggered by environmental exposures, postbronchodilator FEV1 % predicted, postbronchodilator FEV1/FVC < 0.70, RV/TLC and diffusion capacity.

Table 3 shows an overview of the differentiating ability of all relevant items. In Scenario 1 (only medical history questions), eight items were included in the model, four of which showed a statistically significant relationship when differentiating between asthma and COPD: packyears, wheeze, phlegm and allergy. In Scenario 2, ten items were included in the model, six of which showed a significant relation in differentiating between asthma and COPD: age, wheeze, breathlessness, allergy, FEV1 % predicted and FEV1/FVC. In Scenario 3, twelve items were included in the model, six showing statistical significance when differentiating between asthma and COPD: age, wheeze, breathlessness, allergy, FEV1 predicted and FEV1/FVC. Independent of the scenario, postbronchodilator FEV1/FVC was an important discriminant.

**Table 3:** Differentiating abilities of relevant items and overall model performance.

		Scenario 1			Scenario 2			Scenario 3		
Subsection		Asthma	COPD	*p*^m^	Asthma	COPD	*p*^m^	Asthma	COPD	*p*^m^
Medical history questions	Age	0.97 (0.94, 1.01)	1.03 (1.00, 1.06)	0.096	0.96 (0.92, 0.99)	1.05 (1.01, 1.09)	0.014	0.93 (0.88, 0.97)	1.08 (1.03. 1.13)	0.003
	Gender (female)	x^l^			x			x		
	Packyears^a^	0.97 (0.95, 0.99)	1.03 (1.01, 1.06)	0.015	0.98 (0.96, 1.00)	1.02 (1.00, 1.05)	0.10	x		
	Wheeze^b^	2.76 (1.33, 5.57)	0.36 (0.17, 0.75)	0.007	3.62 (1.52, 8.59)	0.28 (0.12, 0.66)	0.004	2.79 (1.15, 6.75)	0.36 (0.15, 0.87)	0.023
	Phlegm^c^	0.33 (0.12, 0.90)	2.99 (1.11, 8.08)	0.030	x			x		
	Breathlessness^d^	x			2.60 (1.05, 6.40)	0.39 (0.16, 0.95)	0.038	2.55 (1.01, 6.46)	0.39 (0.15, 0.99)	0.049
	Ever respiratory allergy^e^	6.97 (3.38, 14.35)	0.14 (0.07, 0.30)	<0.001	4.37 (2.01, 9.50)	0.23 (0.11, 0.50)	<0.001	5.47 (2.49, 11.99)	0.18 (0.08, 0.40)	<0.001
	Respiratory problems^f^	x			x			x		
Lung function tests available to primary care	FEV1 % predicted ECCS^g^				1.07 (1.03, 1.10)	0.94 (0.91, 0.97)	<0.001	1.08 (1.04, 1.11)	0.93 (0.90, 0.96)	<0.001
	FEV1/FVC^h^ < 0.70				0.14 (0.04, 0.52)	7.25 (1.92, 27.45)	0.004	0.11 (0.03, 0.44)	8.81 (2.27, 34.18)	0.002
Lung function tests available to secondary care	RV/TLC^a^							1.06 (0.99, 1.14)	0.94 (0.88, 1.01)	0.096
	Diffusion capacity^i^							x		
Model performance	Explained variance^j^	0.41			0.54			0.56		
	AUC^k^ (95%CI) *p*-value for difference between AUCs	0.84 (0.78–0.89) –			0.89 (0.84–0.93) 0.020^n^			0.89 (0.85–0.94) 0.967^o^		

Odds ratios (95% confidence intervals) for diagnosing asthma or COPD together with corresponding *p*-values are calculated for the three different scenarios based on the items available.

*AUC* area under the curve, *ECCS* European community of coal and steel, *FEV1* forced expiratory volume in 1 s, *FVC* forced vital capacity, *ROC* receiver operator characteristics, *RV* residual volume, *TLC* total lung capacity.

^a^Packyears were missing in 2 subjects, RV/TLC in 3 subjects; there were no further missings.

^b^Wheeze with or without breathlessness (in previous 12 months).

^c^Phlegm after getting out of bed (in previous 12 months).

^d^Breathlessness on exertion (in previous 12 months).

^e^Allergic to pollen, animals, dust mites or seasonal symptoms.

^f^Respiratory symptoms triggered by cold air, smoke or (exhaust)fumes.

^g^Postbronchodilator FEV1 as % of predicted value.

^h^Postbronchodilator FEV1/FVC.

^i^Diffusion capacity in mmol/kPa/min.

^j^Nagelkerke R square.

^k^AUC of ROC curve with COPD as reference group.

^l^‘x’ refers to variables manually removed from the model as *p*-values were >0.10.

^m^For the difference between asthma and COPD diagnoses within each scenario separately.

^n^For the difference between Scenarios 2 and 1.

^o^For the difference between Scenarios 3 and 2.

**Fig. 3:**
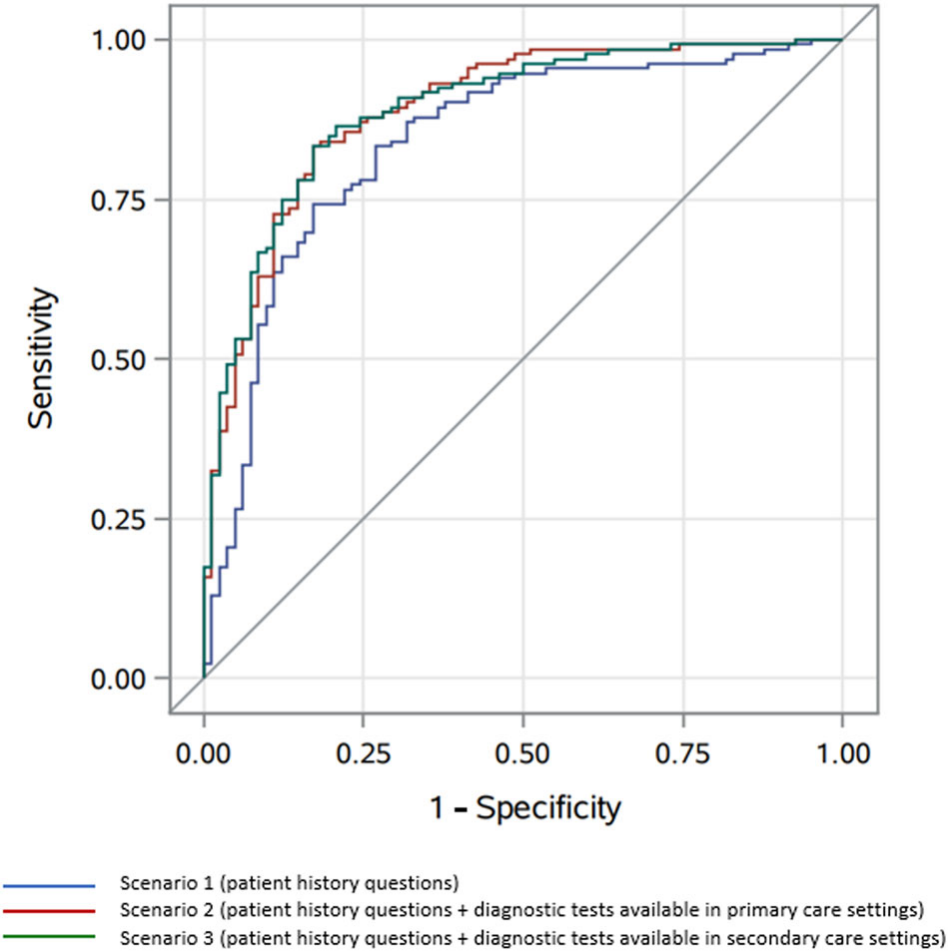
Receiver operating characteristic (ROC) curves for the three scenarios* to differentiate between asthma and COPD diagnoses (*n* = 222). *Area under the curve (AUC) values and *p*-values of comparison between Scenario 1 and 2 and Scenario 2 and 3: see Table 3.

In Scenario 1 the logistic model showed a percentage explained variance of 41% and ROC characteristics showed an area under the curve (AUC) of 0.84 (95% confidence interval (CI): 0.78–0.89)) (Fig. 3). By adding diagnostic variables available to primary care (i.e., spirometry) in Scenario 2, the explained variance increased to 54% and AUC increased to 0.89 (95% CI 0.84–0.93). Finally, by adding more advanced diagnostic tests available to secondary care in Scenario 3, the explained variance increased to 56% but AUC remained 0.89 (95% CI 0.85–0.94). Statistical testing showed a statistically significant difference between the AUCs of Scenarios 2 and 1 (*p* = 0.020) but no such difference between the AUCs of Scenarios 3 and 2 (*p* = 0.967, see Table 3).

## Discussion

In this study, we looked at which patient characteristics distinguish between patients diagnosed with asthma or COPD, and established the added value of spirometry and of more advanced lung function measurements when differentiating between the two chronic airways diseases. Although asthma and COPD are both heterogenous conditions with multiple overlapping features, there are important clinical differences as well. We observed that in the scenario using only medical history questions, it is already possible to reliably distinguish between asthma and COPD. The most important factors to aid differentiation are smoking behaviour, certain respiratory symptoms and the presence of allergies. The use of postbronchodilator spirometry provided important additional discriminative power in correctly labelling a patient as having asthma or COPD. More advanced diagnostic tests that are mainly used in secondary care, such as measuring bronchial hyperresponsiveness and diffusion capacity, did not provide a better differentiation in this primary care study population.

In the present study, both bronchodilator reversibility and bronchial hyperresponsiveness had a similar prevalence in patients diagnosed with asthma and COPD. This finding is noteworthy, as the current GINA guideline refers to reversibility testing and bronchial hyperresponsiveness as criteria supporting the diagnosis of asthma^1^. However, our finding is not unique as previous studies have concluded that solely the presence of reversibility or bronchial hyperresponsiveness does not distinguish between the two obstructive airways diseases^33–36^. Besides these similarities, there were several clinical features that were statistically different between the two diagnostic subgroups and for that reason, these features can aid primary care clinicians when differentiating between asthma and COPD. Using only medical history questions in the logistic model (Scenario 1) already showed rather good differentiating ability (AUC = 0.84). These findings are in line with other studies that assessed the ability of solely using medical history questionnaires to distinguish between asthma and COPD. Beeh et al. concluded that with only medical history questions, it is possible to distinguish between asthma and COPD for the majority of patients with suspected or established obstructive lung disease^37^. Likewise, in their study Tinkelman et al. reported that a simple self-administered questionnaire can facilitate differentiation between obstructive lung diseases^38^. However, these studies did not look at the additional use of spirometry or more advanced diagnostic tests to discriminate between asthma and COPD nor did they quantify this in, for instance, an area under the curve analysis like we did. In the present study we found that postbronchodilator spirometry was important when differentiating the two conditions and together with medical history questions, the discriminating ability of the model improved (from AUC = 0.84 in Scenario 1 to AUC = 0.89 in Scenario 2). In contrast, more advanced diagnostic tests did not provide a better diagnostic differentiation (AUC remained 0.89 in Scenario 3). This does not mean that these tests are useless, as they have an important role in evaluating the presence and severity of structural lung damage (like, for instance, in emphysema and bronchiectasis) and in differentiating obstructive lung disease from other aetiologies in selected patients^39,40^.

A particular strength of our study is that we used standardized methods to conduct the lung function testing and to obtain the respiratory diagnoses. All questionnaires and lung function tests were standardized and prospectively collected, were supervised by certified lung function technicians, and the lung function tests met established quality standards.

Given the central role of general practice in the Dutch healthcare system, nearly all inhabitants are registered in a general practice of their own choice. Therefore, the subjects who participated in the initial DIMCA program and provided for the sample in the current analysis can be seen as representative for the adult Dutch population. On top of this, our study is original in categorizing diagnostic variables based on their availability in different healthcare settings.

However, there were limitations as well. We only looked at the diagnosis itself and did not consider the severity of the diagnosed chronic airways diseases. Because each subject was initially assessed by only one of the chest physicians we were not able to look at the interobserver agreement. Subjects who were considered to have no asthma or COPD were not mutually discussed by the chest physicians to reach a maximum substantiated outcome. However, given that the aim of our study was to differentiate between asthma and COPD and not to distinguish between being ‘respiratory healthy’ or not, we do not consider this to be a relevant limitation of the study.

In some cases the chest physicians’ assessment may have led to false positive diagnoses of COPD, as some subjects who had a post-BD FEV1/FVC value >0.70 (*n* = 3; see Table 2) or reported to never have smoked (*n* = 27) were assigned a COPD diagnosis nonetheless. Unfortunately, we cannot in retrospect ascertain the chest physicians’ specific considerations for assigning this diagnosis in these cases.

Whereas the data collection and diagnostic approach in the DIMCA study by Albers et al.^22^ were conducted in a prospective manner, our study was retrospective in design and we were limited to using a pre-existing list of diagnostic items. The data collection dates from more than a decade ago and therefore several more recent diagnostic tests were not included. For instance, several recent studies have shown that the underlying type of inflammation in patients with asthma and COPD is markedly different^3,41^. Tests like sputum cell count, peripheral eosinophil count, serum IgE and fractional exhaled nitric oxide (FeNO) provide relevant information about the underlying inflammatory process and could support differentiation between asthma and COPD, but were not assessed in our study. Besides their differentiating potential, these inflammatory markers could have taught us more about the pathogenesis of ‘Asthma-COPD Overlap’ (ACO), which is the subject of ongoing debate^42,43^. We call for researchers to perform a similar study as ours in a heterogenous sample of appropriate study subjects, with the addition of the aforementioned contemporary inflammatory markers to the study protocol.

By using the two distinct diagnoses (i.e., asthma and COPD) our study does not increase knowledge on how to identify patients with ACO. However, as the majority (i.e., two-thirds or more)^6^ of patients with chronic obstructive airways disease do not concern ACO, our observations do add insight into how to discriminate between these two diagnoses in a substantial part of the overall group of patients with chronic obstructive airways disease. In other words, the study does not ‘solve’ the wider problem of how to distinguish patients with ACO from those with an ‘unambiguous’ diagnosis of asthma or COPD, but it does contribute to the issue of how to diagnose and distinguish the patients in which there is no overlap.

Lastly, it is important to note that the subgroups of patients labelled with asthma or COPD are defined by the diagnostic criteria used by Albers et al.^22^. These criteria were based on GOLD and GINA guidelines from 2006 and 2007, respectively^26,27^. But despite new pathophysiological insights, the definition, description and diagnostic criteria of asthma and COPD have not substantially changed ever since^1,2^. The fact that we did not apply the current Global Lung Function Initiative (GLI) references values nor the lower limit of normal definition of airway obstruction^44^ will not have had a significant impact on our findings, as this mainly influences the interpretation of presence or absence of obstruction in elderly subjects^45^ who were hardly present in our middle-aged study sample. Thus, in our view using the older guideline-based classification does not render the results of the present study obsolete or invalid.

A final limitation of the study that should be mentioned is that younger adults (i.e., those aged 18–30) were not included in the study. However, as the aim of the study was to differentiate between asthma and COPD and a diagnosis of COPD below the age of 30 is highly unlikely, we do not think this has had a relevant impact on the findings as reported.

Besides the good discriminating ability of solely using anamnestic questions, our results emphasize the importance of postbronchodilator spirometry in distinguishing asthma from COPD and vice versa. However, it is important to realise that the lung function tests in our study were conducted by well-trained staff in a pulmonary function laboratory and interpreted by experienced chest physicians. To translate these results to the real-life setting, it requires standardized procedures, quality assurance and trained clinicians to interpret the spirometry data accurately and this may be difficult to achieve in primary care^46–48^. However, previous studies have shown that it is feasible to conduct reproducible and clinically meaningful spirometry tests in primary care and that primary care clinicians can interpret spirometry test results correctly^29,49,50^. Even while in our study bronchial hyperresponsiveness testing did not improve diagnostic differentiation, it has been shown that bronchial challenge testing is safe and feasible in a suitably equipped primary care diagnostic centre^51^. Referral to secondary care is indicated in the few cases in which it is not possible to establish a diagnosis on the basis of thorough medical history taking and well-conducted spirometry alone, or to exclude other possible underlying conditions.

In conclusion, primary care clinicians should be able to reliably differentiate between asthma and COPD with the combination of relevant patient history questions and postbronchodilator spirometry tests for the majority of patients with suspected chronic airways disease. More advanced diagnostic tests used in hospital care settings do not seem to provide a better overall diagnostic differentiation between asthma and COPD in primary care patients. Given the important additional role of postbronchodilator spirometry in this process of differentiating, the implementation of quality-assured spirometry testing and sufficient training should be mandatory in primary care practices. Furthermore, the availability of inflammatory markers in primary care could potentially provide better discriminating diagnostic ability but we did not investigate this in the current study.

## Supplementary information

Reporting Summary

## Data Availability

The data from the DIMCA study are not made publicly accessible because the variable names, labels and codebook are all in the Dutch language. The dataset can be requested from the corresponding author without restrictions, in which case relevant variables and labels will be translated to English.

## Code availability

Readers can access the code (i.e., SPSS syntax) of the statistical analyses by sending a request to the corresponding author.
